# Interfacial Liquid Water on Graphite, Graphene, and 2D Materials

**DOI:** 10.1021/acsnano.2c10215

**Published:** 2022-12-12

**Authors:** Ricardo Garcia

**Affiliations:** Instituto de Ciencia de Materiales de Madrid, CSIC, c/Sor Juana Inés de la Cruz 3, 28049Madrid, Spain

**Keywords:** interfacial water, graphite−water interfaces, 2D materials-water interfaces, solid−liquid interfaces, AFM, three-dimensional AFM, airborne contaminants, WCA, molecular dynamics simulations

## Abstract

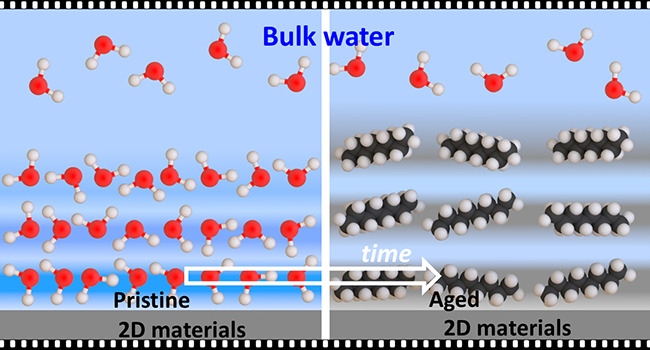

The optical, electronic, and mechanical properties of graphite, few-layer, and two-dimensional (2D) materials have prompted a considerable number of applications. Biosensing, energy storage, and water desalination illustrate applications that require a molecular-scale understanding of the interfacial water structure on 2D materials. This review introduces the most recent experimental and theoretical advances on the structure of interfacial liquid water on graphite-like and 2D materials surfaces. On pristine conditions, atomic-scale resolution experiments revealed the existence of 1–3 hydration layers. Those layers were separated by ∼0.3 nm. The experimental data were supported by molecular dynamics simulations. However, under standard working conditions, atomic-scale resolution experiments revealed the presence of 2–3 hydrocarbon layers. Those layers were separated by ∼0.5 nm. Linear alkanes were the dominant molecular specie within the hydrocarbon layers. Paradoxically, the interface of an aged 2D material surface immersed in water does not have water molecules on its vicinity. Free-energy considerations favored the replacement of water by alkanes.

## Introduction

The characterization and understanding of the optical, electronic, and mechanical properties of graphene, 2D, and few-layer materials have generated an extraordinary scientific activity.^1−5^ The interfacial water properties of graphite-like and 2D materials have also drawn considerable scientific interest.^6−17^ The interfacial water structure on those surfaces has implications on a variety of topics ranging from biosensing^2^ to water desalination^15,16^ and from tissue engineering^5^ to energy storage.^17^

In fact, the understanding of the solid–water interface at the molecular-scale has challenged the scientific community since the discovery of the hydration layers by Israelachvili and Pashely.^18,19^ It is sobering and perplexing to realize that, well into 21st century, the structure of interfacial water on solid surfaces is a source of discoveries and controversies, among them, the hydrophobic effect,^20−23^ the viscosity of nanoconfined water,^24−30^ or the structure of the electric double layer.^31−39^

The interaction of liquid water with graphite-like and 2D materials has generated scientific topics, among them, the wetting transparency of graphene,^6−10,40−42^ the water-flow in nanofluidic channels and nanopores,^14,43−46^ or the ubiquitous presence of organic adsorbates at the graphite–liquid-water interface.^7,11−13,47−52^

**Figure 1:**
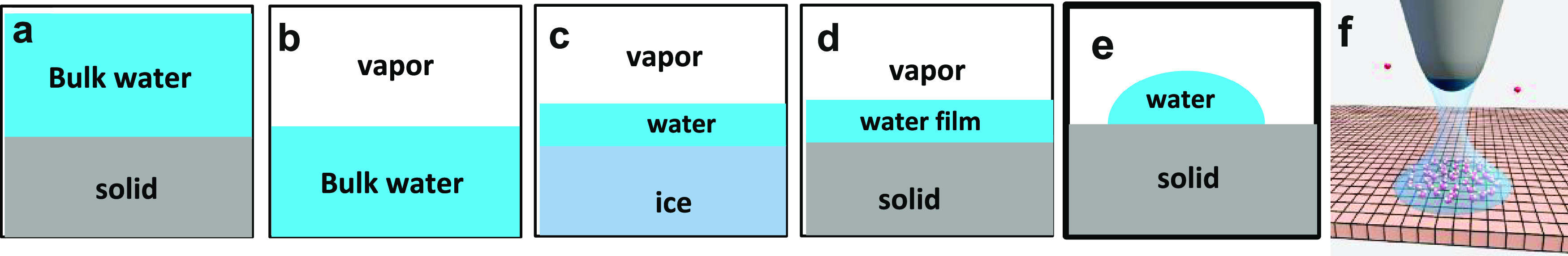
Schemes of the main solid–water interfaces: (a) solid–liquid water, (b) liquid–vapor, (c) liquidlike layers on ice, and (d) thin water film. (e) Drop on a solid surface. (f) Water nanomeniscus bridging two solid surfaces. Panel f adapted with permission from ref (54). Copyright 2021 American Chemical Society.

The study of solid–water interfaces poses several challenges. First, there are several water interfaces (Figure 1). Second, the experimental methods have limitations in terms of sensitivity and/or spatial resolution. Third, it is not always possible to compare high-spatial resolution images with spectroscopy data.

A complete investigation of the solid–water interface requires the characterization of four regions: (1) the solid interface in contact with the water (contact layer), (2) the 1–2 nm deep region above the solid surface where the properties of the water are different from the bulk liquid. In the presence of electrolytes, this region includes the electric double layer and (3) the bulk liquid. (4) The region underneath the solid surface that is influenced by the presence of water. Currently, there is no a single experimental method that can be applied to characterize all the above interfaces.

This context has motivated the development, improvement, or implementation of several microscopy,^53−56^ spectroscopy,^57−61^ and other surface sensitivity^62−65^ methods to characterize the properties of interfacial water on solid surfaces. Some recent reviews provide an updated introduction to the methods and models developed to study solid–liquid interfaces.^62,66−74^ In particular, Björneholm *et al*. review^69^ provides a comprehensive and highly readable introduction to the key topics of water at interfaces. Fenter and Lee’s brief account on the organization of interfacial hydration layers, based on X-ray reflectivity data, provides an insightful introduction to solid–water interfaces.^62^

Until the development of the three-dimensional atomic force microscope (3D AFM),^53^ the experimental methods lacked the spatial resolution and/or sensitivity to reveal with molecular detail the three-dimensional structure of liquid water in the region within 2 nm from the solid surface. The stacking of atomic-scale resolution 2D images (*xy* planes) obtained at different *z* distances from a mica surface showed how the structure of the water changed with the distance to the mica.^75^ Since then, the technique has been applied to study the interaction of liquid water with graphite,^12,49,50,54,76−79^ graphene,^12^ hexagonal boron nitride,^50^ WSe_2_,^12^ and MoS_2_^12^ surfaces.

This review discusses the most recent data on the structure of liquid water in the vicinity of graphite, graphene, and 2D materials surfaces. Atomic-scale resolution images were analyzed in combination with molecular dynamics simulations, nanofluidic channels, vibrational spectroscopies, X-ray reflectivity, and conventional atomic force microscopy data.

In short, on a pristine graphite or 2D materials surface, the interfacial water structure was characterized by the formation of 1–3 hydration layers. The stacking of water molecules in planes parallel to the solid surface reflects changes in the mass density distribution of water across the interface. The water density oscillates around its bulk value with a spatial periodicity of ∼0.3 nm. However, hydration layers on graphite-like and 2D materials surfaces were found to be short-lived.

Many applications of graphite and 2D materials involved a processing step, which required the exposition of the surface to ambient air. Exposure to ambient air was associated with the adsorption of airborne contaminants. Under those conditions, the hydration layers were replaced by 1–3 hydrocarbon layers. Hydrocarbon layers, mostly composed of straight-chain alkanes, were characterized by a spatial periodicity of ∼0.5 nm.

## Key Concepts on Interfacial Water on Surfaces

**Figure 2:**
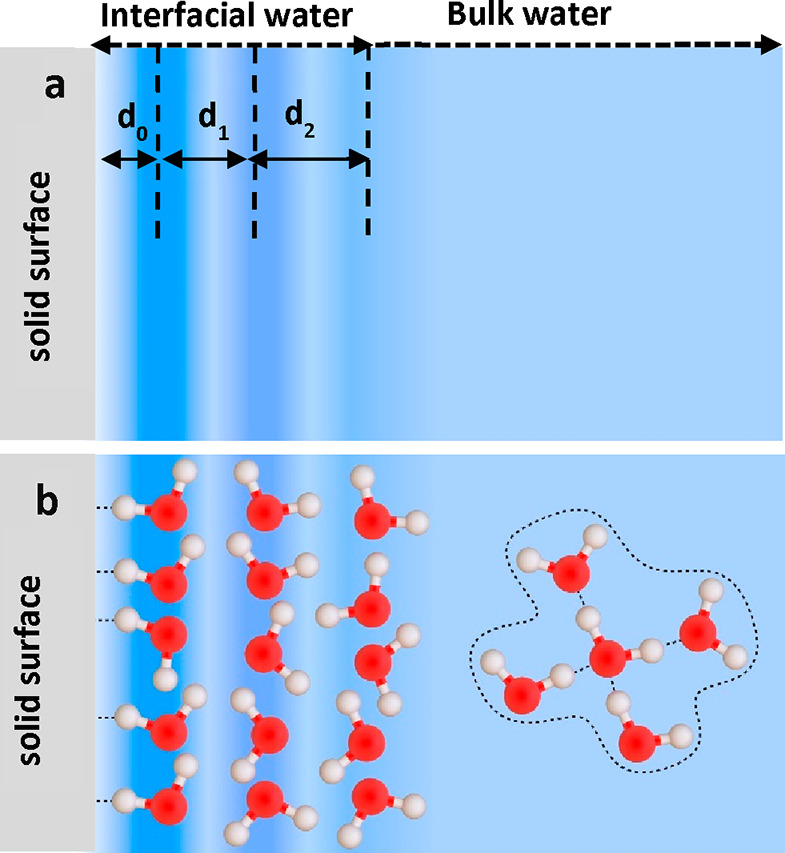
Schematic representation of hydration layers. (a) Out-of-plane (*xz*) profiles of the water density; *d*_0_ is the distance between the 1st liquid layer to the solid surface; *d*_1_ and *d*_2_ are the liquid interlayer distances. (b) Scheme of the water molecules within the hydration layers. Far from the surface, the water adopts a local tetrahedral configuration (bulk water).

Figure 1 describes some of the most common water interfaces, (a) bulk liquid water on a solid surface, (b) a liquid layer on ice, (c) liquid–vapor, and (d) a thin water film adsorbed on a solid surface under ambient relative humidity. The last interface might have two variations, (e) a drop of water and (f) a capillary neck connecting the two surfaces. Furthermore, a solid–water interface has three different regions (Figure 2), (1) the water molecules directly interacting with the solid (contact layer), (2) the 1–2 nm region above the solid where the water density oscillates (interfacial liquid water), and (c) the bulk water.

### Graphite-Like Surfaces

It includes graphite, graphene, and few-layer graphene. 3D AFM experiments performed on graphite, graphene, or few-layer graphene revealed strong similarities on an interfacial liquid water structure.^12,50^ For that reason, here, the results obtained on a given surface, say few-layer graphite, were extrapolated to the other surfaces (graphene, bulk graphite). The same assumption was applied to the other layered materials such as hexagonal boron nitride (hBN), WSe_2_, MoS_2_, and few-layer variants of the last three materials.

### Interfacial Liquid Water

It is defined as the region near a solid surface where the structure and properties of water might be different from those of bulk water. For example, the density distribution profile might be higher or lower than bulk water.^53,62^

The interaction of liquid water with many crystalline surfaces leads to the formation of a few hydration layers (1–3). Hydration layers are separated by ∼0.3 nm, this is, a value close to the average O–O separation in water (0.28 nm).^53,54,62,69^ The separation between layers was not uniform, usually *d*_1_< *d*_2_. For aqueous solutions, the extension of the interfacial liquid region perpendicular to the solid surface depended on the salt concentration. It might extend to 2 nm above the solid surface.^75^

### Liquid Water

It is water obtained by a water purification instrument (ultrapure water). Ultrapure water is characterized by a resistivity of 18 MΩ cm. That value corresponds to an ion concentration of less than 0.04 ppm. Ultrapure water might contain dissolved gases (N_2_, O_2_, CO_2_)^80−82^ and trace amounts of volatile organic compounds,^82^ among them, linear hydrocarbons (alkanes). The concentration of N_2_ and organic contaminants in water might be estimated from the ideal gas and Henry’s laws.^13,82^ At room temperature, the concentrations for N_2_ and linear hydrocarbons are, respectively, 5.5 × 10^–4^ molecules/nm^3^ (0.512 mmol L^–1^ or ≈15 ppm) and 3.5 × 10^–7^ molecules/nm^3^ (≈10 ppb ≡10 μg/m^3^).

### Hydrophobic Surfaces

There is not an accepted scale to quantify surface hydrophobicity. Several parameters, among them, the contact angle, the water adhesion tension, or the free energy of hydration, were proposed to quantify hydrophobicity.^21,83−85^ Estimations of the hydrophobicity of a surface are commonly derived from the values of the static water contact angle (WCA) θ.^83^ Thus, a surface is said to be hydrophobic when 120° ≥ θ ≥ 90°; weakly hydrophobic when 90° > θ > 60° 90°; and weakly hydrophilic when 60° > θ > 0°. A fully hydrophilic surface is considered to have a WCA equal to 0°. The majority of water contact angles reported for graphite, graphene, MoS_2_, hBN, WSe_2_, and similar surfaces were above 55°.^6−10,51,86^ Therefore, 2D materials surfaces should be considered partially or mildly hydrophobic. However, within the 2D materials community, graphite-like surfaces are commonly considered mildly hydrophilic.^85^

### Volatile Organic Compounds

Volatile organic compounds (VOCs) of both natural and human origin are present in air.^87−90^ They might be generated from polymeric materials, fossil fuel combustion, or human breadth.^91^ The most common VOCs are hydrocarbons with typical per-species concentrations of less than 50 μgm^–3^ or equivalently ≤10 ppb.^88−90^ However, the very low concentration of VOCs might be offset by their high affinity to graphite-like surfaces^92−94^ and, in particular, to the graphite–water interface.^52^ VOCs adsorbed on graphite-like surfaces are called airborne contaminants.

Hwang’s group^11^ and Martinez-Martin *et al*.^95^ provided AFM observations on the adsorption of airborne organic contaminants on graphitic surfaces. Those observations were followed by high-spatial resolution AFM measurements characterizing the evolution of graphite and some transition metal dichalcogenide (TMDC) surfaces exposed to ambient air.^96−102^

### Pristine and Aged Surfaces

A graphite, graphene, or a few-layer transition metal dichacolgenide surface is considered pristine when it meets two requirements: (1) it has been freshly prepared (cleaved or otherwise) and (2) immersion in pure water showed the presence of hydration layers separated by about 0.3 nm.

An aged surface is defined as a freshly prepared surface that was exposed to ambient air for more than 60 s. Aging is characterized by the accumulation of physiosorbed hydrocarbon molecules on some regions of the surface. AFM images showed that those adsorbates might form stripe patterns covering micrometer-size regions^50,96−100^ or small nanometer-size islands.^101,102^ Hydrocarbon adsorption probably happens at shorter exposition times (less than 60 s) but data were not available.

Additional evidence on the adsorption of VOCs on graphite-like and few-layer TMDCs surfaces came from water contact angle measurements. It was shown that the WCA of graphene,^7,9,104^ graphite,^7,48^ mono and few-layer hBN,^86^ WS_2_,^106^ MoS_2_,^105^ and InSe^51^ surfaces increased upon exposure to laboratory air. Fourier-transform infrared spectroscopy showed a correlation between an increase in the WCA and the appearance of methylene stretching peaks (at 2930 cm^–1^), indicating the presence of linear alkanes.^7,48,51,86,105,107^ Ellipsometry,^105^ XPS,^51,86^ polarization contrast microscopy,^108^ and electrochemical impedance spectroscopy^109^ data either confirmed or were consistent with the presence of hydrocarbons on graphite-like surfaces upon their exposure to air.

### Wetting

The wettability of a solid surface measures its affinity to water. Wetting might be defined as the attractive interaction of the water molecules with a solid surface. Several theoretical simulations showed that the free energy of adsorption of water on graphene and graphite surfaces was negative.^110−112^ Therefore, water molecules are readily adsorbed on those surfaces. However, under common working conditions, the wettability of a graphite-like surface depends on intrinsic and external factors. The intrinsic factors are related the properties of the substrate such as the crystallographic orientation or the doping properties. The external factors might include the presence of airborne organic contaminants. Some methods were suggested to limit or slow down the adsorption of airborne organic contaminants;^113−115^ however, additional evidence supporting their effectivity was not provided. In fact, recent experimental data suggest that the presence of organic contaminants on graphite, graphene, and 2D materials surfaces upon exposure to air^51^ or water might be unavoidable.^52^

## Methods to Study Interfacial Water on Graphite and 2D Layered Materials

Several techniques have been applied to study the interaction and properties of water with graphite and 2D materials surfaces. Chiefly, among them were water contact angle,^6−10,41,42,103−105^ X-ray reflectivity,^61,116,117^ electron microscopy,^118−121^ vibrational sum-frequency-generation spectroscopy,^42,122−124^ X-ray spectroscopies,^60,125,126^ AFM methods,^127−130^ 3D AFM,^12,49,50,52,54,75−79^ and impedance methods.^48,105,108,109^ The experimental techniques were complemented by a variety of theoretical and molecular dynamics methods.^13,110−113,131−135^ Nanofluidic channels made from two-dimensional crystals enabled the fabrication of devices that relied on the interfacial liquid water properties.^14,46^ Those devices were also applied to study the interaction of water with graphene and hexagonal boron nitride surfaces.^65,136^

### Water Contact Angle

WCA experiments are very popular and useful to characterize the wettability of surfaces under relevant environmental conditions. WCA has been extensively applied to characterize the wettability of graphene,^6−10,137,138^ 2D materials surfaces,^51,86,105,106^ and graphite.^7,103^ The contact angle is a macroscopic observable that reflects a competition between wetting and droplet cohesion.

Water contact angle values on graphite, graphene, and 2D materials surfaces were very sensitive to the condition of the surface, such as the properties of the supporting substrate, the size and type of crystallographic orientations, or the presence of contaminants. Schneider and co-workers made an extensive compilation of WCA values obtained on monolayer graphene either suspended or deposited on different substrates.^10^ The values ranged between 42° for freestanding graphene to 105°. Factors such as the doping of the graphene, the number and the type of defects originated during growth and/or transfer processes, or the adsorption of airborne contaminants were proposed to explain the numerical discrepancies obtained by different groups.^7−10^ MD simulations performed by different groups for graphene showed a large dispersion of values from 45.7°^139^ to above 90°.^140,141^ A model was proposed to explain the changes of the WCA on graphene as a function of the hydrocarbon coverage.^142^

It is important to underline that WCA measurements did not provide direct information on the interfacial liquid water structure. The interfacial structure was inferred by MD simulations, which reproduced the experimental WCA values.

### Transmission Electron Microscopy (TEM)

**Figure 3:**
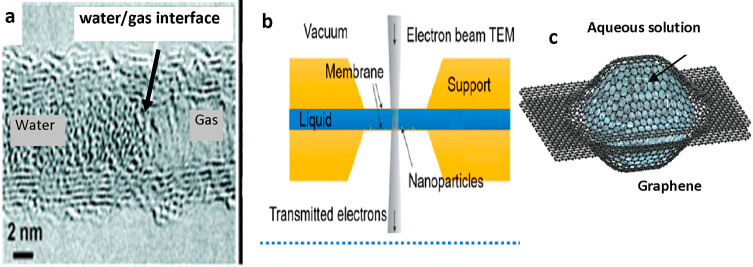
(a) TEM image of water inside a carbon nanotube. Adapted with permission from ref (118). Copyright 2004 American Chemical Society. (b) Schematic diagram of a liquid cell TEM setup. Reprinted with permission from ref (121). Copyright 2022 American Chemical Society. (c) Scheme of a graphene liquid cell. Two graphene membranes encapsulate a liquid solution.

An early TEM experiment showed the capability to image water inside carbon nanotubes of 2–5 nm in diameter.^118^ The high-spatial resolution images resolved the multiwalled carbon nanotube structure (Figure 3). However, the TEM images neither resolved the molecular-scale structure at the surface nor the interfacial liquid structure inside the carbon nanotube. The development of specific liquid cells for electron microscopy enabled the observation of particle nucleation and growth.^55,121,143,144^ Atomic-scale resolution images of nanoconfined water between two graphene layers were interpreted as “square ice”, a phase of water having a symmetry different from the conventional tetrahedrally geometry of hydrogen bonding between water molecules.^119^ Other authors attributed the observed structure to contamination by salt crystals.^145^

The use of graphene and graphene oxide membranes in liquid cells has increased the spatial resolution for imaging nucleation and crystal growth process in liquid.^143,144^ However, liquid-phase TEM lacks the spatial resolution to resolve the molecular-scale structure of solvation layers.

### X-ray Reflectivity (XRR)

In XRR, an incident beam of highly penetrating and high-brilliance X-rays is directed to the solid–water interface. The scattering intensity (observable) is analyzed in terms of the electron density. In general, the electron density of the interface is the physical quantity to be determined. To determine it, the intensity of the reflected beam is compared to the intensities obtained by using parametrized models of the interfacial structure.^62^ The capabilities of XRR to determine the interfacial water structure were tested by imaging the hydration layers on muscovite mica surfaces.^146,147^

Regarding the graphene–water interface, a XRR experiment showed the layering of water on an epitaxial graphene surface.^116^ The XRR data showed a primary hydration layer at a distance of 0.31 nm above the graphene surface. Another hydration layer was observed at 0.6 nm followed by a featureless profile corresponding to the bulk water phase. This result has yet to be reproduced by another XRR experiment. An experiment performed on chemical vapor deposition (CVD) graphene on SiO_2_/Si in water did not observe the layering of water molecules. Instead the data showed the presence of a diffusive layer adjacent to the graphene surface.^117^ The extension of the diffusive layer decreased after the graphene surface was kept immersed in water for 24 h at 25 °C. The existence of a diffusive layer and its dependence on the time the surface was immersed in water suggested the presence of surface contaminants. That interpretation was in line with several WCA and 3D AFM observations on aged graphenic surfaces.

The energy and intensity requirements of the XRR beams demand the use of synchrotron radiation sources. The use of a synchrotron limits the repeatability of the measurements. It restricts also the number and type of solid–liquid interfaces that might be characterized.’

### X-ray Spectroscopies

Many of the methods applied in surface science to characterize surfaces^67^ lack the penetrating capabilities to go through the liquid phase to reach the solid–liquid interface. Salmeron and co-workers developed an experimental equipment to characterize solid–liquid interfaces by using soft X-rays, which do not require synchrotron facilities.^59,60^ In particular, the development of a liquid cell which enabled an X-ray adsorption spectroscopy characterization of electrochemical reactions on graphene surfaces immersed in an aqueous electrolyte.^125^

X-ray photoemission spectroscopy was applied to measure the surface potential of silica nanoparticles dispersed in aqueous electrolytes^35^ and the potential drop in the electrolyte.^36^ However, similar studies involving graphene or 2D materials surfaces immersed in water were not reported. The method lacked the sensitivity to probe directly the structure of solvation layers.^126^

### Vibrational Sum-Frequency Generation

**Figure 4:**
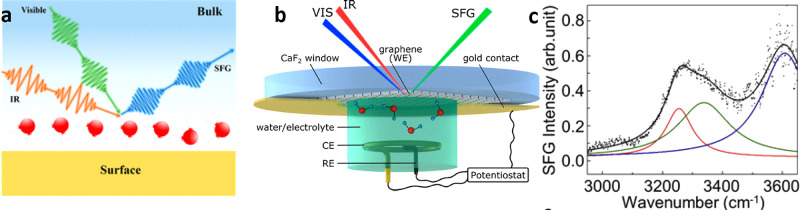
(a) Scheme of a solid–water interface in a vibrational sum-frequency-generation spectroscopy experiment. Reprinted with permission from ref (74). Copyright 2022 American Chemical Society. (b) Scheme of an electrochemical liquid cell for VSFG experiment. Reprinted with permission from ref (122). Copyright 2019 American Chemical Society. (c) VSFG spectrum of water at the water-multilayer graphene (six layers). The spectrum is fitted with three components with center frequencies of ∼3200, ∼3400, and ∼3600 cm^–1^. The broad peat at 3200 cm^–1^ originates from the OH-stretching modes of hydrogen-bonded water molecules at the graphene surface. Reprinted with permission from ref (42). Copyright 2021 Elsevier.

Vibrational sum-frequency generation (VSFG) is an optical spectroscopy method that probes molecular vibrations that take place at interfaces, air–liquid, liquid–liquid, or solid–liquid.^57,58,148^ In VSFG, infrared and visible light pulses are focused on the solid–water interface (Figure 4a,b). Resonant IR excites vibrational modes while the visible light pulse causes an anti-Stokes scattering process. The interaction of the incoming pulses with the interface generates an optical signal with a frequency equivalent to the sum of the IR and visible signals. VSFG is a second-order nonlinear spectroscopy, which requires symmetry breaking to generate a signal.

VSFG was applied to determine the water transparency of graphene.^42,122^Figure 4c shows a VSFG spectrum obtained on a multilayer graphene–water interface. The spectrum shows three bands. Each band was fitted with a Lorentzian function with center frequencies, ∼3200, ∼3400, and ∼3600 cm^–1^. Cho and co-workers^42^ explained the above spectrum as follows. The lower band is associated with OH-stretching modes of H-bonded molecules at the graphene–water interface. The upper band was associated with the dangling OH groups pointing toward the graphene (Figure 4c). Singla *et al*. showed that graphene behaves like a hydrophobic (or negatively charged) surface, leading to enhanced ordering of water molecules at the surface.^123^ It should be noted that the interpretation of the VSFG spectra at graphitic–water interfaces is still under debate.^122−124^

VSFG lacks lateral spatial resolution but the symmetry breaking, which happens naturally at solid–water interface, enables to characterize the orientation of water molecules and the hydrogen-bonding network at the contact layer.

### Atomic Force Microscopy

**Figure 5:**
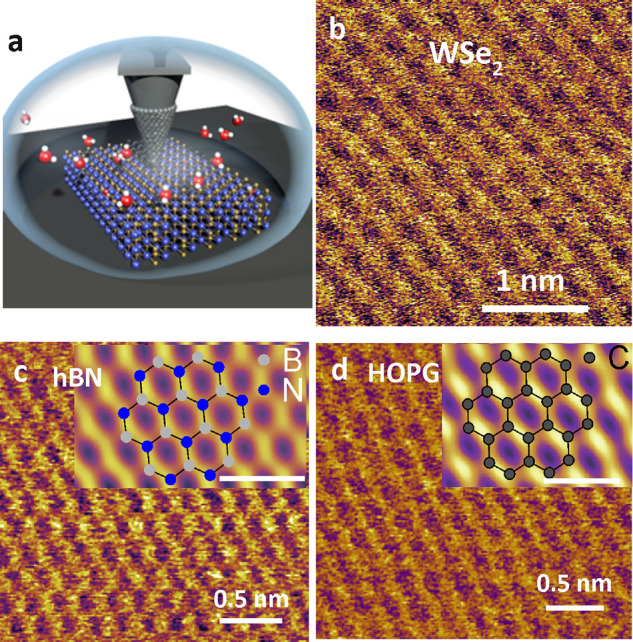
(a) Scheme of AFM imaging a 2D materials surface immersed in water. (b) Atomic-resolution AFM image of a few-layer WSe_2_ material in water. Reprinted with permission under a Creative Commons Attribution 4.0 International License from ref (12). Copyright 2019 Springer Nature. (c) Atomic-scale resolution image of a hBN surface immersed in water. In the inset, nitrogen and boron are depicted, respectively, in blue and gray (scale bar of 0.5 nm). (d) Atomic-scale resolution image of an HOPG surface immersed in water. The inset shows an image with the honeycomb model of graphite (scale bar of 0.5 nm). All AFM phase images. Panels c and d reprinted with permission from ref (50). Copyright 2021 Royal Society of Chemistry.

In AFM, a sharp tip is displaced across the sample surface (Figure 5a). An image of the surface topography is obtained by recording the variation of one or several observables that characterize the tip’s deflection as a function of its *xyz* position on the surface. In contact AFM or in a quasistatic AFM measurement, the tip’s deflection is the main observable. In dynamic AFM modes, the amplitude, the phase shift, or the frequency shift are the main observables.^149,150^

The AFM observables depend on the spatial coordinates. Those changes are associated with changes of the tip–surface force. Atomic-scale resolution images might be generated if the tip’s apex ends in one or a few atoms. Figure 5b–d shows atomic-scale resolution AFM images of WSe_2_, hBN, and graphite surfaces immersed in water.

AFM has been extensively applied to characterize the properties of the graphite-like and 2D materials in a vacuum,^151^ air,^96−102,152−154^ or liquid.^155,156^

In the context of solid–water interactions, early studies were focused on the influence of the relative humidity on the surface topography of graphite surfaces.^157,158^ The wetting properties of graphene and 2D materials were studied by measuring the adhesion force as a function of the relative humidity.^159,160^ Several studies combined experimental measurements and simulations to understand the influence of water molecules and electrolytes on the friction and wear properties of 2D materials.^28,133,161−164^ Other processes such as the self-assembly of organic molecules,^165,166^ the characterization of chemical reactions,^167^ or the evolution of air nanobubbles on graphite surfaces immersed in water were reported.^168,169^

Heath and co-workers developed an ingenious approach to study water confined between 2D materials surfaces.^170,171^ A 2D materials layer (capping layer) was deposited onto a solid substrate (mica, graphite, TMDCs). The capping layer confined the water molecules or films, which were previously adsorbed onto the substrate. The AFM was used to image the surface topography of the 2D layer as a function of the relative humidity. The AFM tip was placed on the 2D crystal face exposed to the air. Topographic variations were interpreted in terms of wetting/dewetting stages,^172,173^ the number of confined water layers,^174,175^ or local charge variations.^176^ Strelcov *et al*. expanded the capabilities of this method by capping an aqueous electrolyte volume with a graphene membrane and performing Kelvin probe force microscopy measurements.^177^

The above method had some limitations to characterize solid–water interfaces. First, it provided indirect images of the solid–water interfaces. The water was never in contact with the tip. The tip and the water were separated by the 2D materials layer. Second, the interpretation of the data might be affected by surface contamination. In fact, Rabe’s group showed that adhesive tapes, which were often used to mechanically exfoliate graphenes onto solid substrates, might have induced some of the ice-like structures previously interpreted as wetting/dewetting processes.^178^ Third, the capping method was unsuitable study processes involving liquid water. For those reasons, this method is not recommended to characterize 2D materials–water interfaces.

### Other Experimental Methods

Other methods were applied to study graphite and 2D materials surfaces immersed in water. In some cases, the experimental setup posed considerable limitations to perform extensive studies of solid–liquid interfaces. In other cases, the method lacked the sensitivity and/or spatial resolution to provide information on the water structure at the molecular level. For example, the existence of hydration layers was revealed by measurements performed with a surface force apparatus (SFA).^63,64^ However, it has been hard to apply the SFA to measure interfacial water on 2D materials. The most common configuration of this instrument makes use of crossed cylindrical silica disks with mica glued onto them. A recent development of the SFA enabled to study the ion mobility within a water gap mimicking a graphene nanopore.^179^ Scanning electrochemical probe microscopy methods were applied to study the electric double layer and catalytic activity of TMDC nanosheets in electrolyte solutions.^180−183^ Those methods provided information on the surface charge distribution but lacked atomic-scale spatial resolution. On the sample surface, the spatial resolution was limited by the size of the probe (∼50 nm).

Scanning probe microscopes operated in an ultrahigh vacuum and at low temperatures were applied to study the adsorption of individual water molecules on metallic and ionic crystal surfaces (submonolayer coverages).^155,184^ Those experiments provided information on specific adsorption sites for water molecules. However, the structure and properties of interfacial water in a vacuum can be very different from those of the liquid water.

Attenuated Fourier-transform infrared spectroscopy (ATR-FTIR) identified the presence of alkanes on graphite-like surfaces exposed to air,^48,51,86,105,185^ but this method cannot be applied to study solid–water interfaces.

### Nanofluidic Channels

**Figure 6:**
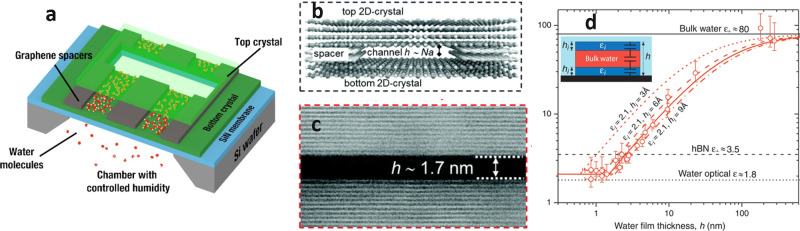
(a) Schematic representation of a nanofludic channel device. (b) Scheme of a channel displaying the top, bottom and spacer layers, with channel height *h*, *N* is the number of layers of graphene spacer, and *a* is the interlayer distance in graphite. (c) Cross-sectional TEM dark field image of a five-layer channel. The nanochannel has a width of 1.7 nm. Horizontal bright lines represent individual layers of graphite. Panels b and c reprinted with permission from ref (115). Copyright 2021 Royal Society of Chemistry. (d) Dielectric constant measurements of a nanochannel as a function of the thickness (*h*). Symbols: ε_⊥_ is the dielectric constant perpendicular to the solid–water interface. The *y*-axis error is the uncertainty in ε_⊥._ Red curves: Calculated ε_⊥_(h) behavior for the model sketched in the inset. It assumes the presence of near-surface layer with ε_i_ = 2.1 and thickness *h*_i_, whereas the rest of the channel contains the ordinary bulk water. Panel d reprinted with permission from ref (65). Copyright 2018 American Association for the Advancement of Science.

In 2016, Geim and co-workers^14^ introduced a method to fabricate nanofluidic channels with subnanometer control in the channel height (2D nanoslits). The height was controlled by using graphene layers as spacers between the top and bottom layers of the channel (Figure 6a–c). Those devices were primarily designed to study water transport properties in nanocapillaries.^136,186^ The water properties measured in ultrathin channels (say sub-2 nm in height) might be strongly influenced by the structure of the water on the walls of the nanochannel. Therefore, some experiments involving nanofluidic channels were designed to offer insight into some general properties of nanoconfined water such as the regimes of superfast water transport,^186^ the dielectric constant of nanoconfined water,^65^ or the validity of the macroscopic Kelvin equation to describe capillary condensation at the atomic-scale.^136^

Figure 6d shows the dependence of the dielectric constant perpendicular to the solid–water interface. The dielectric constant decreased from 80 (far from the 2D crystal) to 2 for a separation between top and bottom layers of 1 nm. Fumagalli *et al*. reasoned that the binding of water molecules to the 2D crystal restricted the orientation degrees of freedom of the water molecules at the surface.^65^ An alternative explanation based on the formation of hydrocarbon layers was proposed by Uhlig *et al*. (see below).^50^

### Theoretical Methods and Molecular Dynamics Simulations

Theoretical methods and simulations were essential to interpret the experimental data and, in the process, to advance our understanding of solid–liquid interfaces. It is beyond the scope of the review to introduce the key features of those methods. I opted for selecting some contributions that were applied to describe the interaction of water with graphite and 2D materials surfaces. The methods range from density functional theory approximations^187−190^ to *ab initio* molecular dynamics simulations^124,131,132,191^ and from molecular dynamics simulations based on empirical force fields^192−203^ to a variety of semiclassical methods.^204−212^

First principle or *ab initio* methods are more accurate because they minimize the number of assumptions to model the interactions among water molecules and the water and the solid surface. However, they have a high computational cost, which, in practice, limits the system size to about 10^3^ molecules and the time of the interaction to a few picoseconds. On the other hand, force field molecular dynamics might simulate larger systems over longer times. Some semiclassical methods might describe the system in its final equilibrium state, which facilitates experiment-theory comparisons.

**Figure 7:**
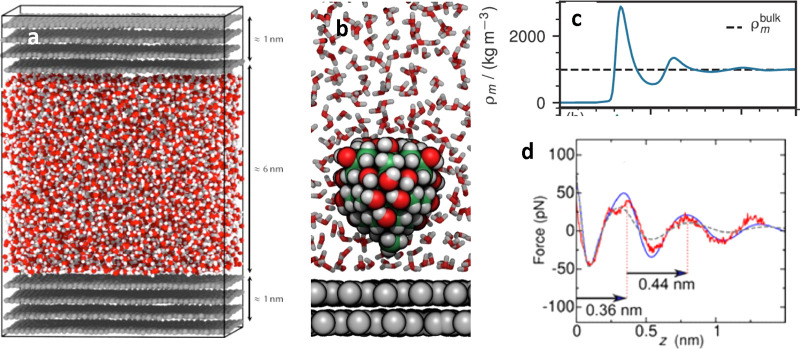
(a) Example of a MD simulation snapshot of water enclosed between two few-layer graphene walls. The simulation frame corresponds to the equilibrated system. Adapted with permission under a Creative Commons Attribution 4.0 International License from ref (78). Copyright 2019 APS. (b) MD snapshots of a model AFM tip asperity near a graphene–water interface. For clarity, only a cross section of the solvent molecules is shown. Atoms are shown as spheres (H, white; graphite C, gray; other C, green; oxygen, red). Reprinted with permission from ref (50). Copyright 2021 Royal Society of Chemistry. (c) Interfacial water density profile within two graphene walls separated by 3 nm. The peaks in the marks the center position of the hydration layers. Adapted with permission from ref (202). Copyright 2020 ACS. (d) Comparison between experimental AFM (blue and gray) and MD (red) data. The MD simulations were performed on a graphene layer immersed in hexane. The AFM curves were obtained on an aged graphite surface immersed, respectively, in water (gray) and in hexane (blue). Adapted with permission from ref (50). Copyright 2021 Royal Society of Chemistry.

Figure 7a shows a MD snapshot of water molecules enclosed between two few-layer graphene surfaces.^78^Figure 7b shows a MD snapshot of an AFM tip (hydroxylated diamondoid cluster) immersed in water near a graphene surface.^50^Figure 7c shows the oscillations in the water density profile near a graphene surface (MD simulation),^202^ while Figure 7d presents a comparison between AFM data and MD simulations for different graphite–liquid interfaces.^50^ The experimental data represents the force–distance curves obtained on an aged graphite surface immersed in water (gray) and an aged graphite surface immersed in hexane (blue). The MD simulations represents a force–distance curve obtained on a surface of graphene immersed in hexane. The agreement obtained between experiment and theory indicated that straight-chain alkanes were accumulated at the surface of an aged graphite surface immersed in water.

## Three-Dimensional AFM (3D AFM)

**Figure 8:**
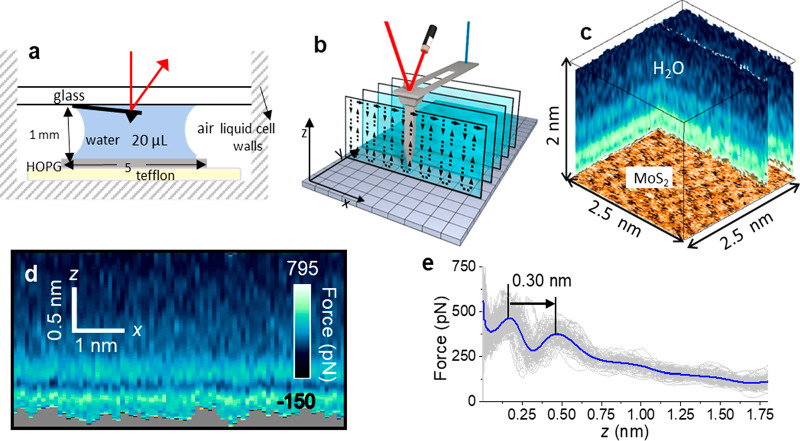
(a) Scheme of a 3D AFM liquid cell and its size. (b) Scheme of the tip’s displacements in 3D AFM. The red laser tracks the tip’s oscillation. The blue laser drives the cantilever tip’s oscillation. (c) 3D AFM image of a MoS_2_–water interface. The 3D image might be split into different 2D maps. An image of the MoS_2_ lattice is shown at the bottom. Reprinted with permission under a Creative Commons Attribution 4.0 International License from ref (12). Copyright 2019 Springer Nature. (d) 2D force (*x*, *z*) map obtained of a graphite–water nanomeniscus interface. (e) Force–distance curves extracted from the 2D force map shown in *d*. The force–distance curves include oscillatory and monotonic terms. The blue line is the average force–distance curve. Two hydration layers are observed. Panels d and e reprinted with permission from ref (54). Copyright 2021 American Chemical Society.

Three-dimensional AFM is a probe-based method in which the three spatial components of the tip displacement with respect to the solid surface *x*, *y*, and *z* are synchronized.^53^ An external force drives the tip at one of its flexural resonances, while the tip is displaced in the volume of liquid (Figure 8a). The frequency of the tip’s oscillation must be several orders of magnitude higher that any of the frequencies associated with the *x*, *y*, or *z* displacements. The tip explores the solid–liquid interface by acquiring a series of *xz* planes, one per each *y* position (Figure 8b). Those planes are combined to generate a volume map of the interface (Figure 8c). The tip’s oscillation might be controlled with either frequency,^213,214^ amplitude,^75^ or bimodal modulation^215^ feedbacks.

Several conditions must be met to generate atomic-scale resolution maps of the interface. The amplitude of the tip’s oscillation must be smaller than the thickness of the solvation layers to be measured. Typical values were of few tens of picometers.^53^ The microcantilever-tip system should be also driven by a method that generates resonant frequency curves compatible with the point-mass model of a driven harmonic oscillator with damping,^150^ for example, photothermal excitation. The latter condition facilitates the transformation of the tip’s observables (amplitude, phase, or frequency shifts) into force values *F*(*x*, *y*, *z*).^216−218^

For low molarity aqueous solutions and uncharged tips,^219^ the force might be associated with atomic-scale changes of the solvent density.^210,220^ In the simplest model to describe the interaction of the tip with a liquid, the solvent-tip approximation, the tip is considered as a single solvent molecule (water), and the force applied to the tip *F*(z) is described by the following equation:^220^
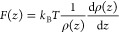
1where *z*, *k*_B_, *T*, and ρ denote distance between the vertical tip position, Boltzmann’s constant, absolute temperature, and water density, respectively. This equation enabled to convert the water density maps obtained from MD simulations into force maps that can be compared with the experimental force maps.

Three-dimensional AFM is currently the only experimental method capable of imaging solid–liquid interfaces with atomic-scale spatial resolution in the three spatial coordinates. In the past few years, 3D AFM has found a wide range of applications. It has characterized the structure of water and aqueous solutions on rigid crystalline surfaces^30,38,79,221−223^ and soft biomolecules.^215,224−226^ Similarly it has been applied to study the solvation layers formed by some organic solvents^227,228^ and ionic liquids^229−231^ on graphite, MoS_2_, and mica surfaces. 3D AFM was also applied to characterize the electric double layer at the graphite–electrolyte interfaces.^219,232,233^

Figure 8d shows a 2D force (*x*, *z*) map obtained inside a nanoscale water bridge connecting a graphite surface and a silicon tip. The corresponding force–distance curves (Figure 8e) show the presence of two hydration layers separated by 0.30 nm.

## Complexity of Liquid Water Interfaces on Graphite and 2D Materials

It is convenient to discuss independently the interaction of liquid water with pristine and aged graphite, graphene, and 2D materials surfaces.

### Working Configurations

Many of the applications foreseen for graphite-like materials and aqueous solutions might involved complex working configurations. Under those configurations, the presence of airborne or liquidborne organic molecules might be unavoidable.

A large number of 3D AFM images were obtained on graphite, graphene, and few-layer TMDCs surfaces immersed in water. Those images showed an interfacial water structure characterized by the presence up to three solvation layers parallel to the graphite surface.^12,49,52,77−79^ The separation between the layers was 0.5 nm (average value). This value was about 0.2 nm larger than the one expected for hydration layers (0.3 nm). MD simulations showed that an interlayer distance of 0.5 nm was incompatible with the presence of hydration layers.^50^ The above observations received two different interpretations based, respectively, on the accumulation of hydrocarbons (alkanes) and dissolved gas molecules (N_2_). Hwang^47^ and Sivan^49^ groups proposed that the layers were originated by the condensation of dissolved gas molecules (mostly N_2_). This explanation was at odds with many experimental results^7,50−52,54^ and MD simulations^13^ (see below). Alternatively, Uhlig *et al*. proposed that interlayer distances of 0.5 nm indicated the presence of hydrocarbon layers.^12,50^

**Figure 9:**
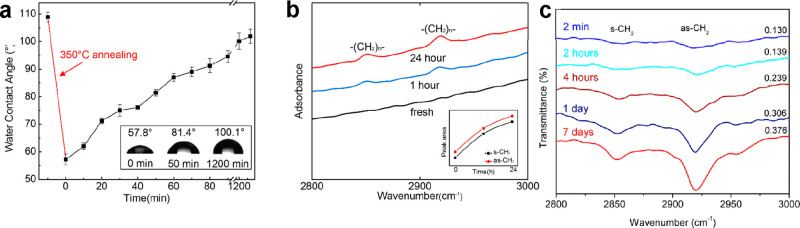
(a) WCA measurement of a 20 nm InSe film on SiO_2_/Si substrate over time after 350 °C thermal annealing. The sample was taken out of the CVD chamber at time 0. (b) FTIR spectra for InSe film as a function of time after air exposure. The spectra reveal a rising volume of organic species −(CH_2_)_*n*_– (2850 and 2930 cm^–1^). Panels and b reprinted with permission from ref (51). Copyright 2020 American Chemical Society. (c) ATR-FTIR of MoS_2_. Sample was exfoliated and aged in air for specified time. Symmetric methylene stretching occurs at 2850 cm^–1^ and asymmetric methylene stretching occurs at 2920 cm^–1^. Reprinted with permission from ref (105). Copyright 2018 American Chemical Society.

Figure 9a–c shows the correlation between WCA and FTIR data as a function of the time in contact with ambient air. An increase of the contact angle, which indicated a higher hydrophobicity, correlated with a FTIR spectra that showed the presence of peaks associated with the stretching of C–H bonds. Similar correlations were also reported for graphene,^7^ graphite,^7,103^ MoS_2_,^105^ or hBN.^86^

Let us describe three experimental results that illustrated the high affinity of organic contaminants toward graphite surfaces immersed in water and the variety of organic contaminant sources.

**Figure 10:**
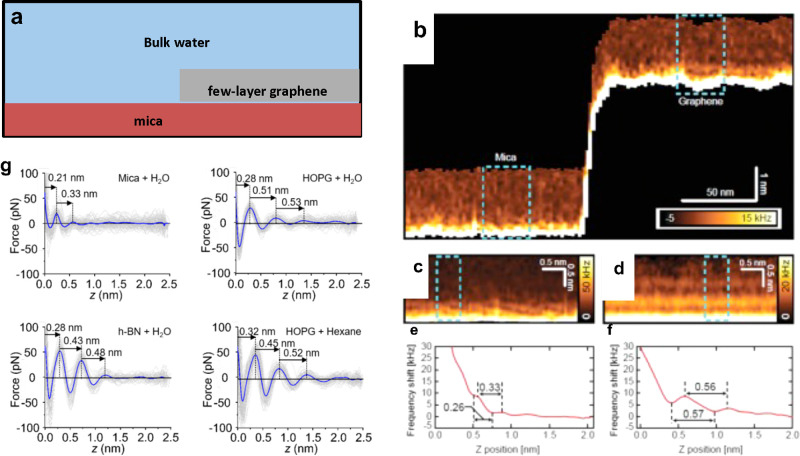
(a) Scheme of a sample immersed in water, which has adjacent mica and graphene regions. (b) AFM topography image. (c) 2D profile map on a mica region (top). The bottom panel shows the force–distance curve averaged over the dashed line region marked in the top panel. (d) 2D profile map on a few-layer graphene region. (e) Force–distance curve averaged over a mica region (dashed line region marked in (c)). (f) Force–distance curve averaged over a few-layer graphene (dashed line region marked in (d)). Panels b–f reprinted with permission from ref (77). Copyright 2018 Royal Society of Chemistry. (g) Force distance curves obtained from top to bottom, left to right on mica, graphite, and hexagonal boron nitride surfaces immersed in pure water. The bottom right panel corresponds to a graphite surface immersed in hexane. The interlayer distance on mica corresponds to hydration layers. The interlayer distances measured on graphite and hBN surfaces are consistent with the presence of hydrocarbon layers. The surfaces were exposed to ambient air for about 5 min before immersion in the liquid. Reprinted with permission from ref (50). Copyright 2021 Royal Society of Chemistry.

Yang *et al*. designed an experiment to measure the interfacial liquid water structure on adjacent mica and few-layer graphene surfaces.^77^ A few-layer graphene flake was deposited on a mica surface. Afterward, the mica containing the graphene flake was immersed in pure water (Figure 10a). The 3D AFM images taken on mica showed layers with a periodicity of 0.3 nm (hydration layers), while on the graphene flake the interlayer distance was about 0.5 nm. (Figure 10b–f). This result indicated, on one hand, the high affinity of a few-layer graphene surface to the presence of trace amounts of organic contaminants dissolved in the water. On the other hand, it showed under identical conditions the striking differences between hydrophilic (mica) and hydrophobic surfaces. The above findings were supported by other 3D AFM experiments performed on graphite, graphene, few-layer MoS_2_, few-layer WSe_2_, and mica surfaces immersed in the same liquid water (Figure 10g).^12,50^ On graphene, graphite, few-layer MoS_2_, and few-layer WSe_2_, the interlayer distances were about 0.5 nm, while on mica the interlayer distances were about 0.3 nm.

Seibert *et al*. AFM images of graphite surfaces immersed in water^129^ showed the formation of stripe domains when plastic syringes were used to inject the water but not when glass syringes were used, suggesting that the domains are composed of organic molecules either native to the plastic or adsorbed to the plastic from ambient air.

Similarly, Berkelaar *et al*.^234^ observed that some objects identified as gaseous nanobubbles in AFM images of a graphite surface did not disappear when exposed to a flow of degassed water for 96 h. They found that the nanobubble-like objects were induced by the use of disposable needles in which PDMS contaminated the water.

### Gibbs Free Energy

**Figure 11:**
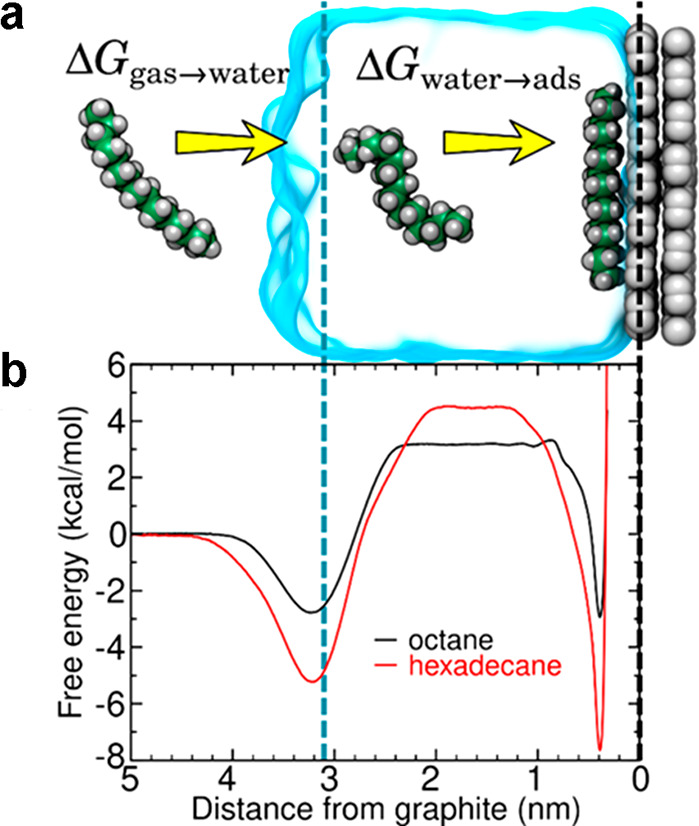
(a) Snapshots from an MD simulation used to calculate the hydration (Δ*G*_gas→water_) and isolated adsorption (Δ*G*_water→ads_) free energies. (b) Free energy for transfer of alkanes from air to overlayers at the graphite–water interface, as calculated from MD simulations. Reprinted with permission from ref (52). Copyright 2021 Royal Society of Chemistry.

Free-energy calculation techniques in the context of MD simulations were applied to understand the thermodynamics of hydrocarbon adsorption.^13,52^ The Gibbs free energy of the process Δ*G*_air→monolayer_ was separated into three components (Figure 11a). Those components were the free energy associated with the hydration of the hydrocarbon molecule (Δ*G*_air→water_), the free energy for adsorption of the hydrocarbon molecule to the graphite–water interface, and, finally, the free energy associated with transfer of the adsorbed, but isolated, hydrocarbon molecule into a hydrocarbon monolayer (Δ*G*_ads→mono_).

Figure 11b shows the free energy curves for the adsorption of two straight-chain alkanes (octane and hexadecane) on graphite. Those hydrocarbons form part of the VOCs detected in indoor air.^87−89^ Theoretical results^13^ have showed that heavy hydrocarbons such as hexadecane form complete monolayers at the graphite–water interface even at trace ambient concentrations in air (∼1–100 μg/m^3^). The free energy profiles showed two local minima, one at the gas–water interface and the other at the graphite surface. The minimum at the gas–water interface indicated that the equilibrium concentration of an alkane molecule was larger at this interface than in bulk air or water. These minima were separated by an energy barrier associated with hydration of such hydrophobic molecules. The free energy at the graphite surface was associated with the lowest free energy, implying that the equilibrium concentration at the graphite–water interface is much higher than the ambient concentration. Moreover, adsorption of alkanes to the graphite–water interface was cooperative. Isolated adsorbed alkane molecules nucleate to form aggregates, further reducing the free energy until a complete monolayer was formed. Overall, the calculations showed that adsorption of alkanes from the gas phase to a graphite surface immersed in water was thermodynamically favorable and therefore spontaneous:
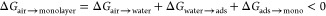
2

### Pristine Configuration

A pristine condition means that the surface, the liquid water, and the surroundings have no trace of organic contaminants. The data described below represents a summary of several experiments performed by 3D AFM on several pristine surfaces. The results showed that the interfacial liquid water structure on pristine conditions was characterized by the presence of up to three hydration layers. Those layers were separated by a distance of 0.3 nm (average value).

To circumvent or avoid altogether the presence of airborne or liquidborne contaminants, Uhlig and Garcia studied the interfacial liquid water structure of nanoconfined water.^54^ To that aim, high-spatial resolution AFM was applied to select a region of a graphite surface free from adsorbates (pristine region). In that region, water vapor molecules were condensed into a water nanomeniscus.^54^ The experiment involved the formation of a nanomeniscus between a sharp AFM tip and a local region of graphite surface (5–10 nm in diameter, 250–300 nm^3^). The process behind the formation of the nanomeniscus, condensation driven by thermodynamics, together with its small size of the nanomeniscus (∼300 nm^3^) meant a graphite–water interface free from airborne contaminants.

3D AFM images showed that the interfacial water structure was characterized by interlayer distances in the 0.3 nm range. That value was in agreement with the distance between the first and the second peak of the water density profile predicted by MD simulations on graphene or graphite surfaces.^94,201,202^ Additional 3D AFM experiments involving larger volumes of water also showed interlayer distances consistent with the presence of hydration layers.^52^ Furthermore, it coincided with the values reported by an early X-ray reflectivity measurement.^116^ Therefore, the existence of hydration layers on pristine graphite surfaces must be considered proven.

**Figure 12:**
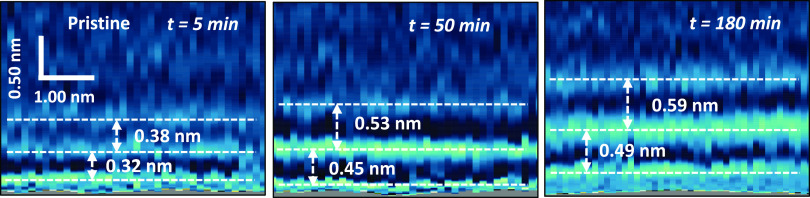
Evolution of the interfacial water structure on graphite: from hydration (left) to hydrocarbon layers (center and right panels). The graphite surface was immersed in pure water at time *t* = 0 s and kept in those conditions for 3 h. The panels represent 2D force (*x*, *z*) maps extracted from 3D AFM volume images. The discontinuous line indicates the average positions of the molecules within a layer. Adapted with permission from ref (52). Copyright 2022 Royal Society of Chemistry.

Arvelo *et al*. implemented a 3D AFM method to follow the evolution of the hydration layers formed on a pristine graphite surface (Figure 12).^52^ The results indicated that hydration layers were initially formed on a pristine graphite surface. However, those layers were replaced over time (30–60 min) by 2–3 layers of alkane-like hydrocarbons. The transition between hydration to hydrocarbon layers was discontinuous. The new interlayer distances were in the 0.45–0.55 nm range.

The experiment did not determine the specific source hydrocarbons. Either the airborne hydrocarbons were adsorbed in some parts of the instrument and later diffused to the graphite–water interface or they entered through the air–water interface. The interlayer distance evolution shown in Figure 12 underlined the difficulties to keep a graphite-like surfaces immersed in pure water under pristine conditions.

**Figure 13:**
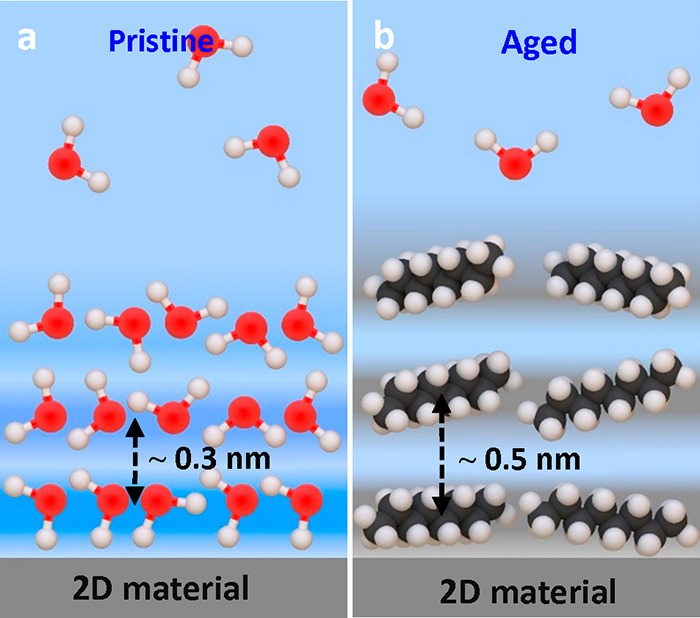
(a) Scheme of interfacial liquid water on graphite and 2D materials under pristine conditions. The interface is characterized by the presence of 1–3 hydration layers (*d*_i_ ∼ 0.3 nm). (b) Same as (b) but under working conditions which in practice means aged surfaces. The presence of airborne or liquidborne organic contaminants gives rise hydrocarbon layers (*d*_i_ ∼ 0.5 nm). Molecules are not drawn to scale.

Figure 13 summarizes the interfacial liquid water structures on graphite and 2D materials surfaces. Under pristine conditions, the interfacial water structure was characterized by the presence of a few hydration layers separated by a distance of 0.3 nm (average value) (Figure 13a). On an aged surface, the interlayer distances were characterized by 0.5 nm (average). Those distances corresponded to the layering of hydrocarbons (alkane chains) (Figure 13b). The hydrocarbon molecules came from the detachment and dissolution of airborne contaminants deposited on the surface during its exposure to ambient air.

A possible pathway for the hydrocarbon layer formation shown in Figure 12 might be as follows. An isolated hydrocarbon molecule dissolved in the water will diffuse to the surface. The high affinity of linear hydrocarbons toward 2D materials will imply that molecule will stay near the surface. Overtime other hydrocarbons will arrive to the surface and cooperative interactions among them will led to the hydrocarbon layers built up. This process leads to the expulsion of the of the water molecules from the 2D materials surface.

## Implications

The presence of hydrocarbon adsorbates on graphite-like and 2D materials surfaces exposed to ambient air is pervasive. Even if the exposition time is very short, say a few seconds, the adsorption of hydrocarbons might be enhanced if the surface is immersed in water. Free energy considerations favor the replacement of water by linear chain hydrocarbons. It is concluded that applications relying on the interaction of 2D materials with an aqueous solution might be affected by the presence of the hydrocarbon layers. Let us re-examine some results obtained on 2D materials and graphite-like surfaces immersed in water by considering the presence of hydrocarbon layers.

Figure 6d shows the capacitance as a function of the separation of liquid water confined between two 2D-crystal surfaces. The data showed that the effective dielectric constant of the interface decreased from 80 at large separations to ∼2 at 1 nm. Fumagalli *et al*. proposed that a value of ε = 2 was caused by a strong interaction happening between the water molecules and the 2D-crystal surface.^65^ This interaction restricted the rotational degrees of freedom of water molecules, which lead to the decrease in ε. However, the presence of hydrocarbon layers at the interface offers an alternative explanation. The dielectric constant of alkane molecules is of ε ≈ 2 at *T* = 295 K. A parallel-plane capacitor model like the one depicted in Figure 6c with the top and bottom capacitors characterized by the dielectric constant of linear alkanes and a thickness of 1 nm will also reproduce the experimental data. MD simulations of water confined between two graphene walls showed^202^ that to reproduce the capacitance measurements in terms of hydration layers required to introduce significant modifications in the width of the water layer.

In the context of carbon-based supercapacitors, Duignan and Shao^235^ noted that carbon materials showed an areal capacitance that was an order of magnitude lower than both that of standard metals and theoretical expectations. Their quantum mechanical calculations showed that the standard explanation of this unusually low capacitance, which was based in terms of the space charge capacitance, was inadequate. They proposed that a layer of hydrocarbon impurities was likely the dominant cause of the low capacitance of graphite. That explanation was in line with the 3D AFM observations.^12,50^ Furthermore, several contributions reported a significant decrease of the double layer capacitance of graphite after immersion in pure water.^48,109,242^

The flow properties of confined water in carbon-based materials or devices have generated unexpected results.^29,237^ Some contributions reported flow rates,^238^ measured for water flow through membranes of carbon nanotubes (CNTs) with diameters of 1.3–7.0 nm, which were two to five orders of magnitude greater than those calculated by the no-slip Hagen–Poiseuille equation. An increased flow of water was also reported when reducing confinement below 2 nm.^14^ However, other contributions reported a decrease in the flow rate in some nanopores.^239^ According to Wu *et al*.^29^ those differences may arise from variations in the strength of the interaction between water and nanopore walls, which strongly depends on the contact angle of water on those walls. WCA experiments showed that the adsorption of hydrocarbons on 2D crystals increased the contact angle.^7,9^

The friction and nanorheological properties of 2D-materials–water interfaces should be influenced by the presence of alkane layers. The dynamic viscosity values of alkane liquids at room temperature^240^ such as hexane (0.313 mPa·s), octane (0.55 mPa·s), or decane (0.9 mPa·s) are smaller than those of water (1.0016 mPa·s). On the other hand, dodecane exhibits a higher viscosity (1.34 mPa·s). In fact, the friction coefficient of few-layer graphene in dodecane was found to be higher than that in water.^163,241^ However, it is not straightforward to predict if this result would also apply for the interfacial water structure formed on 2D materials surfaces. The issue remains unexplored.

### Stripe Patterns of Hydrocarbon Molecules

**Figure 14:**
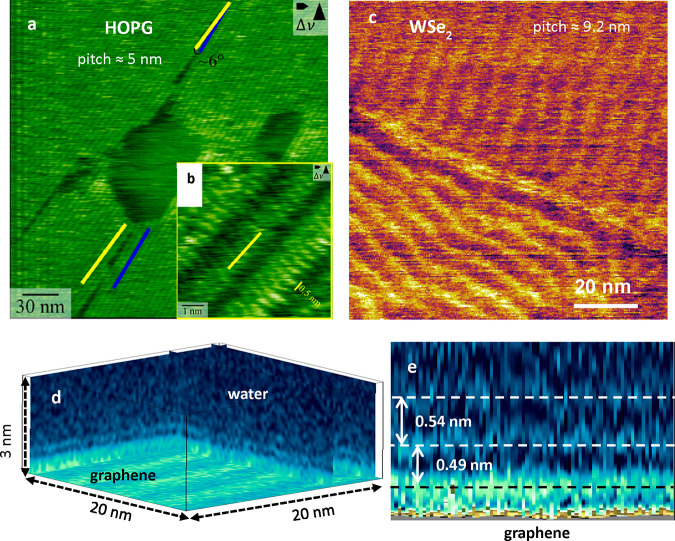
Stripes on HOPG, WSe_2_, and graphene surfaces. (a) AFM image of a stripe-covered graphite surface measured in water. The stripes were formed by the adsorption of liquidborne hydrocarbon molecules. Reprinted with permission from ref (129). Copyright 2020 American Chemical Society. (b) Molecular-scale resolution image of a stripe observed on a HOPG surface immersed in water. The periodicity matches the width of linear chain alkanes. The line indicates the orientation of the stripe (yellow line in a). Adapted with permission from ref (129). Copyright 2020 American Chemical Society. (c) AFM phase image of stripe patterns on a WSe_2_ surface immersed in water. Reprinted with permission under a Creative Commons Attribution 4.0 International License from ref (12). Copyright 2019 Springer Nature. (d) 3D AFM image of a graphene surface immersed in water. The stripe pattern observed on the graphene has a pitch of 5 nm. 2D AFM *xz* force map of the graphene–water interface extracted from the 3D AFM data shown in panel d. Reprinted with permission under Creative Commons BY-NC-ND license from ref (245). Copyright 2020 M.R.Uligh and R.Garcia.

Several AFM studies reported the presence lamellar rows or stripes on graphite-like surfaces exposed to air^96−100^ or immersed in water^11,50,129,236^ (Figure 14a,b). The stripes were also observed on other 2D materials surfaces immersed in water such hBN^50^ and WSe_2_^12^ (Figure 14c). The stripes were arranged in periodic patterns covering up to micrometer squared size regions. Several periodicities from 4 to 10 nm were reported. Those periodicities might come from the presence of different types of straight-chain alkanes. Within a stripe, molecular-scale resolution images showed an arrangement of molecular chains with a periodicity of about 0.5 nm^50,129^ (Figure 14b). That value was very close to the molecular diameter of a straight-chain alkane molecule (0.45 nm). The molecules were oriented perpendicular to the stripe direction. Those patterns were similar to the ones observed by the adsorption of linear alkanes C_*n*_H_2*n*+2_ (*n* = 10, 12, 14, 16) from solution on graphite.^243,244^

On aged graphite, few-layer, or 2D materials surfaces, the hydrocarbons adsorbed on the surface came from the ambient air (airborne hydrocarbons). In general, those patterns remained stable or even grew once the surface was immersed water. In some cases, the stripe patterns observed in air disappeared when the graphite surface was immersed in water. It was shown that the force applied during AFM imaging is a key factor to observe the stripes. The stripes might be removed by increasing the force.^50^ Liquidborne hydrocarbons might also form stripe patterns on pristine graphite surfaces immersed in water.^129^

Figure 14d shows a 3D AFM image of a graphene surface immersed in water.^245^ The graphene surface showed a stripe pattern with a 5 nm pitch. The liquid layers observed on top of the pattern showed interlayers distances of about 0.5 nm (Figure 14e). That finding demonstrated that alkane molecules in both solid (stripes) and liquid (solvation layers) phases were simultaneously observed at graphene–water interfaces.

Hwang *et al*. observed the existence of periodic stripes on graphite surfaces upon immersion in water.^11,242^ The data was interpreted in terms of the adsorption of N_2_ gas molecules. However, there was neither experimental evidence nor theoretical simulations that supported the formation of lamellar rows from the adsorption of N_2_ gas molecules. On the other hand, the formation of lamellar rows of alkane molecules on graphite-like surfaces is a well-established experimental observation.^243,244,246,247^ The lamellar rows observed by the direct deposition of alkanes on graphite surfaces^243,244,247^ were very similar to the ones found on the same surfaces exposed to ambient air^96−99^ or immersed in pure water.^11,50,100,242^

### Interfacial Liquid Water on Hydrophobic Surfaces

The replacement of the water by hydrocarbon molecules on the surface of graphite-like materials was driven by free energy considerations. The same principle should apply to any crystalline hydrophobic surface immersed in liquid water. The specifics of the material would appear in the values of the free energy components. The replacement of water molecules by hydrocarbon layers might also apply to any heterogeneous surface containing polar and nonpolar domains, for example, a protein. However, the nonplanar character of a protein surface and the closeness between polar and nonpolar regions might imply a negligible free energy gain for the adsorption of a hydrocarbon. In fact, theoretical calculations by Comer and co-workers showed that the fee energy of adsorption of small aromatic molecules on the outer surface of a carbon nanotube decreased (absolute value) when the diameter of the nanotube was decreased.^94^

## Conclusion and Outlook

Materials such as graphite, graphene, single and few-layer MoS_2_, WSe_2_, and hBN are categorized as mildly hydrophobic. They exhibit large atomically flat regions that are very well-suited to perform fundamental studies on the interaction of water with materials with technological interest. Several applications in energy storage, tissue engineering, or water desalinization depend on the interaction of aqueous solutions with the surface of 2D materials. Those factors have motivated the application and improvement of several techniques to study 2D materials surfaces immersed in water. In particular, 3D AFM has provided atomic-scale resolution maps of the interface formed by liquid water and a graphite, graphene, and few-layer MoS_2_, WSe_2_, and hBN surfaces. Those images together with molecular dynamics simulations and experimental data from WCA, X-ray reflectivity, 2D nanoslits, electrochemical capacitance, and vibrational spectroscopies enabled a detailed characterization of the 2D materials–water interface. A key finding of the 3D AFM data was the existence of two different interfacial water structures.

Under pristine conditions for the surface and the liquid water, the interfacial water structure on graphite-like and 2D materials surfaces was characterized by the formation of up to three hydration layers. The stacking of water molecules in planes parallel to the solid surface was associated with changes in the mass density distribution. The water density oscillates around its bulk value with a spatial periodicity of ∼0.3 nm.

Most of the applications envisioned for 2D materials and aqueous solutions involve a processing step where the 2D materials surface might get in contact with ambient air. Under those conditions, the interfacial liquid water structure was characterized by the stacking of hydrocarbon molecules. Those layers were separated by a distance of about 0.5 nm. High-spatial resolution images, MD simulations, and spectroscopy data indicated that linear alkane molecules were the dominant species within the hydrocarbon layers. The above conclusions remained valid for the three-dimensional counterparts of the 2D materials described here.

Graphite-like and 2D materials have applications in nanofluidics, energy storage, desalination, water filtration, or tissue engineering. The interfacial water structures reported for graphite and 2D materials will facilitate the understanding of complex solid–liquid interfaces. Such as those characterized by the presence of several electrolytes, molecular species and electrified solid surfaces.

## Vocabulary

2D materials: a crystalline solid made up of a single layer of atoms
3D AFM: a nanomechanical microscope with the capability of resolving the structure of a liquid in the vicinity of a solid surface
airborne contaminant: a volatile organic compound
few-layer materials: a crystalline solid made of a few layers (2–10) of atomic planes
interfacial water: the layer of water molecules within 1 nm from a solid surface
volatile organic compound: any organic compound that is present in ambient air in trace amounts
